# Controllable Synthesis and Catalytic Performance of Gold Nanoparticles with Cucurbit[*n*]urils (*n* = *5*–*8*)

**DOI:** 10.3390/nano8121015

**Published:** 2018-12-06

**Authors:** Liangfeng Zhang, Simin Liu, Yuhua Wang, Haijun Zhang, Feng Liang

**Affiliations:** The State Key Laboratory of Refractories and Metallurgy, Coal Conversion and New Carbon Materials Hubei Key Laboratory, Hubei Province Key Laboratory of Science in Metallurgical Process, School of Chemistry & Chemical Engineering, Wuhan University of Science and Technology, Wuhan 430081, China; wustzlf@163.com (L.Z.); liusimin@wust.edu.cn (S.L.); wangyuhua@wust.edu.cn (Y.W.); zhanghaijun@wust.edu.cn (H.Z.)

**Keywords:** gold nanoparticles, cucurbit[*n*]urils, controllable synthesis, catalysis

## Abstract

A series of gold nanoparticles (AuNPs) was prepared in situ with different cucurbit[*n*]urils (CB[*n*]s) in an alkaline aqueous solution. The nanoparticle sizes can be well controlled by CB[*n*]s (*n* = *5*, *6*, *7*, *8*) with different ring sizes. The packing densities of CB[*5*–*8*] and free surface area on AuNPs were determined. A direct relationship was found between the ring size and packing density of CB[*n*]s with respect to the AuNP-catalyzed reduction of 4-nitrophenol in the presence of NaBH_4_. The larger particle size and higher surface coverage of bigger CB[*n*]-capped AuNPs significantly decreased the catalytic activity. Furthermore, this work could lead to new applications that utilize AuNPs under an overlayer of CB[*n*]s for catalysis, sensing, and drug delivery.

## 1. Introduction

**Figure 1:**
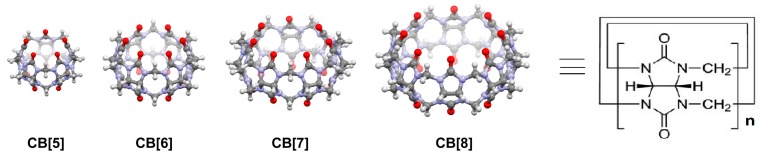
Structures of cucurbit[*n*]urils (CB[*n*]s, *n* = *5*–*8*).

Noble metal nanoparticles with well-defined morphology, composition, and unique physical and chemical properties have already demonstrated their promise in catalysis, sensing, and biomedical applications [1,2,3,4,5]. Although gold and noble metals can be stabilized in different substrates—oxide structures, for example [6,7,8]—these nanoparticles are generally produced from a suitable metal salt in the presence of reducing and stabilizing agents [9,10], which are usually toxic chemicals. Different families of supramolecular macrocycles, including cyclodextrins, calix[*n*]arenes, pillar[*n*]arenes, and cucurbit[*n*]urils, have been employed to direct the synthesis and assembly of noble metal nanoparticles as reducing agents and/or capping agents [11]. Among them, cucurbit[*n*]urils (CB[*n*]s, Figure 1) have attracted particular attention owing to their advantages over other macrocycles, such as their high binding affinity to various organic and metallic cations, their low toxicities, and their ability to assemble themselves in supramolecular nanostructures by capping metal surfaces [12,13,14,15,16,17].

In most cases, chemisorptions, one of the major immobilization methods, has been applied to bring together macrocycles and nanoparticles. This is done by binding bifunctional linkers or using the affinity of thiol-containing molecules for noble metals [11,18,19,20]. As for cucurbit[*n*]urils, a family of macrocyclic cavitands encompassing glycoluril units, the oxygen atoms of the carbonylated ring are responsible for the interaction with metal nanoparticles [21,22,23,24,25,26,27,28] initially reported for flat gold surfaces [29]. The advantage of the electrostatic adsorption of CB[*n*]s on metal is the ease of usage.

The effect of CB[*n*] macrocycles on the structure and stability of in situ prepared metal nanoparticles has been systematically studied on only a few occasions in the case of silver nanoparticles and gold nanoparticles [23,24]. The procedure was based on the use of CB[*5*–*8*] as a stabilizing molecule and borohydride as a reducing agent. Bigger CB[*7*] and CB[*8*] could produce silver nanoparticles, while smaller CB[*5*] and CB[*6*] failed to produce silver nanoparticles. Although CB[*5*–*8*] all could produce gold nanoparticles, CB[*6*] produced the largest gold nanoparticle and CB[*7*] produced the smallest one. No direct relationship was found between the ring size and particle size.

Geckeler et al. developed another environmentally benign and simple procedure to prepare gold, palladium, and silver nanoparticles using CB[*7*] as both a reducing agent and a stabilizing agent in basic media [22,26]. However, researchers did not follow this method to prepare and test CB[*5*–*8*]-capped metal nanoparticles. In this work, CB[*5*–*8*]-capped AuNPs were prepared using a modified procedure in the presence of sodium hydroxide without extra reducing agents. Their solubility was good, and they could be fully re-dispersed in an aqueous environment.

CB[*n*]-capped metal nanoparticles have been reported to have unexpected properties and interesting applications in catalysis, especially in the reduction of 4-nitrophenol (4-NP) by the environmentally friendly sodium borohydride to produce 4-aminophenol (4-AP) [20,22,26,30,31]. The catalytic performance of CB[*n*]-capped metal nanoparticles is still not well understood due to the low number of cases studied. Additionally, most cases were focused on the catalysis of CB[*7*]-capped nanoparticles. Very recently, M. del Pozo and coworkers prepared gold nanoparticles stabilized with CB[*6*], CB[*7*], alpha-cyclodextrin (α-CD), beta-cyclodextrin (β-CD), and highly sulfated-beta-cyclodextrin (HS-β-CD) in the presence of NaBH_4_. In their reduction of 4-nitrophenol, the calculated apparent kinetic constant values (*K_app_*) were related to the gold surface available as active catalytic points [32]. It should be noted that this was the first time that CB[*6*]-capped gold nanoparticles were employed to catalyze 4-NP reduction.

**Scheme 1:**
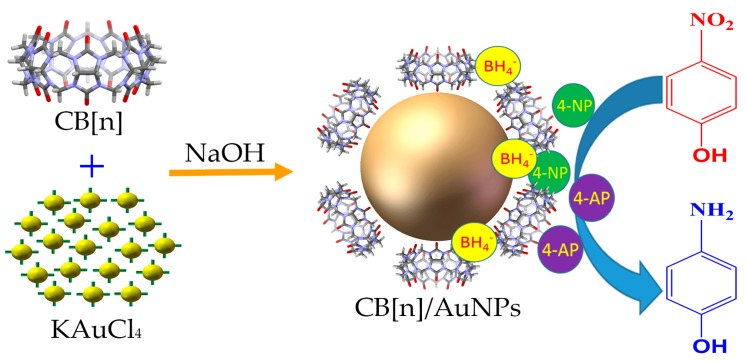
Schematic illustration of the formation of gold nanoparticles (AuNPs) with cucurbit[*n*]urils (CB[*n*]s) in the presence of sodium hydroxide and their application for the catalytic hydrogenation of 4-nitrophenol (4-NP) to 4-aminophenol (4-AP).

Nitrophenol reduction to aminophenol by gold nanoparticles (AuNPs) in the presence of excess sodium borohydride is a “model catalytic reaction” [31,32,33,34,35]. Such a reaction is a pseudo-first-order reaction and can be easily monitored using UV-VIS spectroscopy. It has been widely utilized to test new nanomaterials [32,33,34,35,36,37,38]. In this work, gold nanoparticles capped with the four most readily available CB[*n*]s (*n* = *5*, *6*, *7*, *8*) (CB[*n*]/AuNPs) were employed to catalyze the reduction of 4-NP, and the influences of particle size, CB[*n*]s effect, and packing density were investigated. To the best of our knowledge, this is the first time that CB[*n*] coverage on metal nanoparticles and the reduction of nitro compounds catalyzed by CB[*n*]/AuNPs have been systematically studied (Scheme 1). These results are important for the further development of applications of CB[*n*]-capped nanoparticles or metal surfaces in catalysis and other interesting fields.

## 2. Materials and Methods

### 2.1. Instrumentation

The UV-VIS absorption spectra were recorded on a UV-3600 Shimadzu UV-VIS-NIR spectrophotometer. Transmission electron microscopy (TEM) was performed using a JEM-2100UHR STEM/EDS. X-ray powder diffraction (XRD) pattern was tested on an X’Pert PRO MPD diffractometer with nickel-filtered Cu Ka radiation. A thermal gravimetric analyzer (TGA) STA449 was used for the investigation of thermal properties of the samples. Agilent 600 MHz DD2 spectrometers were used to record ^1^H NMR spectra, and chemical shifts were recorded in parts per million.

### 2.2. Materials

KAuCl_4_ was purchased from SASS Chemical Technology Co., Ltd. (Shenzhen, China), HCl was purchased from Chemical Reagent Factory of Xinyang City, and 2-mercaptobenzimidazole was purchased from Aladdin. Cucurbit[n]uril was prepared by our group, and all other reagents were purchased from Sinopharm Chemical Reagent Co., Ltd. (Shanghai, China).

### 2.3. Synthesis of CB[n]-AuNPs by In Situ Reduction of KAuCl_4_

CB[*n*]-AuNPs were prepared following the reported procedure with some modifications [22]. The same amount of KAuCl_4_ (5 mM) was added to a solution of CB[*n*] (*n* = *6*, *7*, *8*) with 5 mM of HCl (6 mol/L), and 5 mM of CB[*5*] in double distilled water. Yellow precipitate was produced immediately. After the mixture was adjusted to pH = 13~14 by NaOH solution (3.2 mol/L), all solid particles were dissolved and a clear solution was achieved. After standing for 48 h under ambient conditions, the desired CB[n]/AuNPs were produced. It should be noted that some CB[*8*] solids were precipitated after adding NaOH solution, and the solids were washed with 6 mol/L HCl. Finally, all CB[*n*]/AuNPs were washed with double distilled water by means of centrifugation.

### 2.4. Catalytic Reduction of 4-Nitrophenol

A freshly prepared NaBH_4_ solution (0.2 mL, 0.0105 M) was added to a 4-NP solution (0.2 mL, 0.35 mM) in a quartz curve (1 mL). After seconds of stirring, CB[*n*]/AuNPs colloid suspension (0.6 mL, 8.89 mg/L of gold atoms), calculated by inductively coupled plasma-atomic emission spectrometry (ICP-AES), was added and the mixture was monitored using UV-Vis measurements.

## 3. Results and Discussion

**Figure 2:**
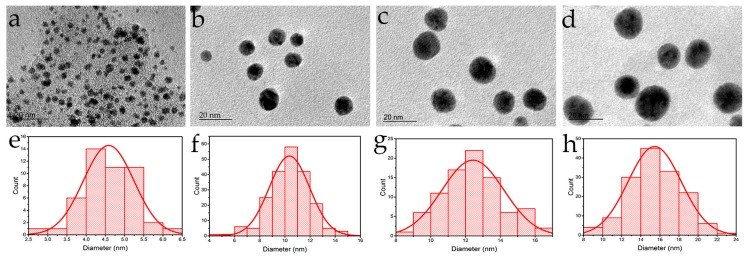
Representative TEM micrographs of AuNPs capped with CB[*5*] in (**a**); CB[*6*] in (**c**); CB[*7*] in (**e**); and CB[*8*] in (**g**). Size distribution plots of AuNPs capped with CB[*5*] in (**b**); CB[*6*] in (**d**); CB[*7*] in (**f**); and CB[*8*] in (**h**).

The morphology and size of the obtained nanocomposites were examined by transmission electron microscopy (TEM). As shown in Figure 2, spherical gold nanoparticles with uniform size distribution were produced by CB[*5*–*8*]. The average diameter of AuNPs increased by 4.6 ± 0.7, 10.4 ± 1.6, 12.4 ± 1.8, and 15.5 ± 2.5 nm, with increasing portal diameters of CB[*n*]s (Table 1). Notably, the ring-size of macrocycles did affect the sizes of nanoparticles in the synthesis, a finding consistent with the results reported by Scherman et al. [24]. Moreover, in our case, CB[*n*]s allowed much better control over size and polydispersity of the AuNPs. The diameter of the portals of CB[*5*], CB[*6*], CB[*7*], and CB[*8*] were 0.24, 0.39, 0.54, and 0.69 nm, respectively. The numbers of C=O units and portal size played a relevant role in arranging CB[*n*]s around the gold surface and thus finalizing the nanoparticle size.

The formation of AuNPs in situ by CB[*n*]s in basic media was established by both UV-VIS spectroscopy and visual detection (color change) (Figure S1). Consistent with previous observations, the surface plasmon band of AuNPs was localized around 530 nm with a diameter of 4–16 nm [39,40], and generally redshifted and broadened because of the formation of self-assembled nanostructures in the presence of CB[*n*]s [21,22,23,24,25,26,27,28]. The nanocomposites obtained with different CB[*n*]s as capping agents showed typical X-ray diffraction (XRD) patterns of AuNPs (Figure S2). The diffraction peaks observed at 38.2°, 43.3°, 64.6°, 77.9°, and 81.8° could be ascribed to the (111), (200), (220), (311), and (222) planes of the Au crystals. The high intensity of the diffraction peak at 38.2° suggests that AuNPs are mostly dominated by (111) facets [22]. CB[*n*]/AuNPs were also analyzed by Fourier transform infrared (FTIR) spectra and compared with pure CB[*n*]. As shown in Figure S3, there was one typical absorption peak around 1730 cm^−1^ of C=O stretching at which CB[*n*] portals could be observed. After capping gold nanoparticles, two C=O stretching bands could be observed. Similar to the typical C=O stretching, C–N stretching bands of CB[*n*]s at 1475 cm^−1^ also underwent a significant redshift after capping gold nanoparticles. These changes further confirmed the portal carbonyl groups interaction with the gold surface [21,22,23,24,25,26,27,28].

In order to quantify the coverage of CB[*n*]s on AuNPs, CB[*n*]/AuNPs were subjected to TGA analysis. Figure S4 shows the TGA curves of CB[*n*]s and as-prepared AuNPs. In this experiment, the CB[*n*]/AuNPs were heated to 800 °C at 5 °C/min under nitrogen, and the change in the weight of the samples was recorded. The CB[*n*]s packing densities on AuNPs as a function of ring size were roughly calculated from the weight fraction of CB[*n*] with respect to the AuNPs core, determined by TGA and TEM (Table 2) [41,42]. It could be calculated that the typical CB[*n*] coverage is around 0.08 molecules/nm^2^, 0.97 molecules/nm^2^, 1.04 molecules/nm^2^, and 1.38 molecules/nm^2^ for CB[*5*]-, CB[*6*]-, CB[*7*]-, and CB[*8*]-capped AuNPs, respectively. A more detailed calculation is addressed in the Supplementary Materials. The surface coverage is understandable and agrees with the results reported previously—more carbonyl oxygens and macrocyclic effects may benefit the binding affinity of CB[*n*] towards the metal surface, and the portals of the larger CB[*7*] and CB[*8*] may find a suitable arrangement and better adapt to the surface of AuNPs [23]. The CB[*5*] coverage is much smaller than the value for bigger CB[*n*]s, which in our case suggests the importance of the presence of six or more C=O units in bigger macrocyclic rings.

**Figure 3:**
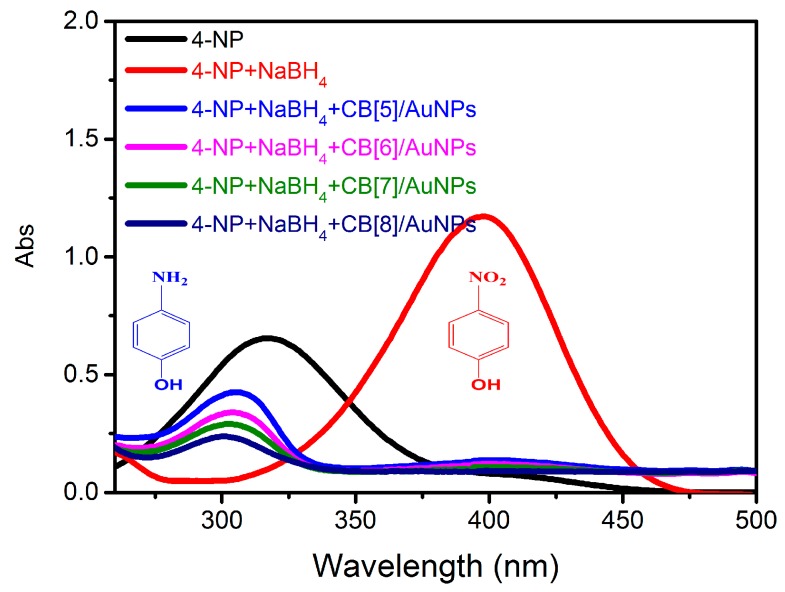
UV-VIS spectra of 4-NP reduction catalyzed by CB[*n*]/AuNPs at 25 °C.

After the CB[*n*]/AuNPs were characterized, the reduction of 4-NP by nanocomposites was carried out and monitored by UV-VIS spectroscopy. A typical absorption band shifted from 317 nm (corresponding to 4-NP) to 400 nm (corresponding to the 4-nitrophenolate ion) after the addition of NaBH_4_ (Figure 3). Only after the addition of AuNPs colloidal suspension did the 4-nitrophenolate band decrease, with the appearance of a new peak at 305 nm (corresponding to 4-AP, a product of the reduction reaction). The rate constant was obtained by fitting the data from Figure S5. Citrate-capped AuNPs were also calculated for comparison. The rate constants for the CB[*5*]/AuNPs, CB[*6*]/AuNPs, CB[*7*]/AuNPs, and CB[*8*]/AuNPs were 11.55, 7.17, 5.98, and 1.95 min^−1^, respectively (Table 2). Although all CB[*n*]/AuNPs exhibited excellent catalytic activity, these rate constants were lower than those of the citrate-capped AuNPs with similar size, which were 13.39 and 7.22 min^−1^ for 4-nm and 16-nm AuNPs. Although no experimental data proved the inclusion between CB[*n*] and 4-NP [30,31], the catalysis of CB[*5–7*] was also evaluated, excluding CB[*8*] because of its low solubility. The rate constants for CB[*5–7*] (Figure S6) were 0.13, 0.11, and 0.10 min^−1^ in the presence of NaBH_4_. These were very close to the rate constant of NaBH_4_ alone (0.08 min^−1^) and one or two orders of magnitude lower than that of CB[*n*]/AuNPs. These results suggest that CB[*n*] has no significant effect on the kinetics of 4-NP reduction by CB[*n*]/AuNPs, since this reaction is known to require a catalyst and usually a metal NP [43,44].

**Scheme 2:**
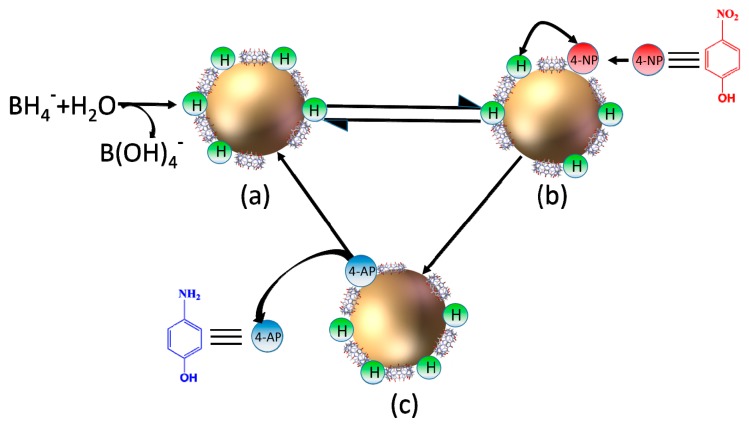
The proposed mechanism for the reduction of 4-NP to 4-AP catalyzed by CB[*n*]/AuNPs in the presence of NaBH_4_.

It has been proposed that the 4-NP reduction on the AuNP surface follows a Langmuir−Hinshelwood mechanism, where reactants initially adsorb on the nanoparticle surface and gold assists the electron transfer [30,31,32,33,34]. In the case of our CB[*7*]-AuNPs, for a constant concentration of 4-NP, a faster reaction was achieved when the NaBH_4_ concentration was augmented. High 4-NP concentrations hindered the NaBH_4_ access to the surface and slowed down the substrate degradation (Figure S7). This suggested that both reactants competed for a limited number of sites on the surface, and the reaction rate was governed by the free surface area (catalytic active sites) available for the reaction and diffusion. The proposed mechanism can be found in Scheme 2. In step (a), the active hydrogen and B(OH)_4_^−^ are formed after the hydrolysis of NaBH_4_. The active hydrogen is then transferred to the CB[*n*]/AuNPs and adsorbed at the gold surface in step (b). In step (c), the active hydrogen at the gold surface reacts with 4-NP, resulting in the product 4-AP.

**Figure 4:**
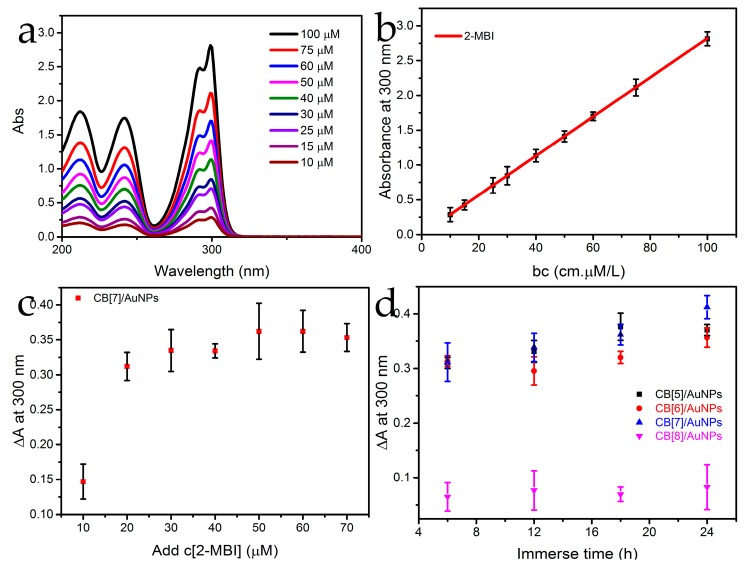
Adsorption experiments of 2-mercaptobenzimidazole (2-MBI) under 25 °C. (**a**) UV-VIS spectra of 2-MBI at different concentrations; (**b**) fitting plot for absorbance coefficient of 2-MBI; (**c**) concentration-dependent plot for CB[*7*]/AuNPs saturated with 2-MBI for 24 h; (**d**) time-dependent plot for CB[*n*]/AuNPs saturated with 2-MBI (100 μM).

As discussed in the previous works, the induction time is dictated by a substrate-induced surface restructuring that is necessary to render the metal nanoparticles an active catalyst [45,46]. As shown in Table 2, as the amount of CB[*n*] on the gold surface increases, the induction time increases. This follows the trend that a more electron-rich gold surface (i.e., fewer places are occupied by capping agents) activates the NaBH_4_ more easily and thus requires less induction time [47,48]. Therefore, the available surface area not passivated by CB[*n*]s on AuNPs was calculated using 2-mercaptobenzimidazole (2-MBI) in the following work. Adopting an upright conformation on AuNPs, 2-MBI was of similar size as 4-NP and 4-AP, and was reasonably used to estimate the correlation between the rate constant and free active site density for 4-NP reduction [49,50]. The absorbance coefficient of 2-MBI at 300 nm was determined to be 28,150 cm^−1^ M^−1^, which is slightly different from the reported 27,400 cm^−1^ M^−1^ [51]. After incubation for 6 h with 100 μM of 2-MBI, CB[*n*]/AuNPs were saturated with 2-MBI. Based on the 2-MBI adsorption data, the nominal 2-MBI saturation packing densities on CB[*5*]/AuNP, CB[*6*]/AuNP, CB[*7*]/AuNP, and CB[*8*]/AuNP were determined to be 5.78, 6.68, 9.02, and 2.36 nmol/cm^2^, respectively (Figure 4). It has been estimated that bigger macrocycles cover larger gold surface areas by TGA. Therefore, the extra 2-MBI absorption of CB[*6*]- and CB[*7*]-AuNPs could be attributed to the host–guest complexation between CB[*6*] or CB[*7*] with a benzimidazole derivative [52,53,54], which was also determined by our NMR study (Figure S8). As for CB[*5*] and CB[*8*], without a strong interaction with 2-MBI [12,13,14,15,16,17], the free active sites on the CB[*n*]/AuNP decreased as the CB[*n*] ring size increased. In avoiding the complexation between 2-MBI with CB[*n*], and because of the strong binding affinity of amantadine towards CB[*n*] (Figure S9) [14], a mixture of 2-MBI and amantadine (1:1 molar ratio) was used to detect the reactive surface sites. The nominal 2-MBI saturation packing densities on CB[*5*]/AuNP, CB[*6*]/AuNP, CB[*7*]/AuNP, and CB[*8*]/AuNP were determined to be 2.89, 2.14, 2.02, and 1.27 nmol/cm^2^, respectively. Because the thiol group of MBI is a much stronger ligand to gold and could possibly displace the CB[*n*] from the AuNPs surface, a mixture of adenine (which could lie flat on the gold surface with a much lower binding affinity to gold) and amantadine (a 1:1 molar ratio) was used to detect the reactive surface sites (Figure S9) [50]. The nominal adenine saturation packing densities on CB[*5*]/AuNP, CB[*6*]/AuNP, CB[*7*]/AuNP, and CB[*8*]/AuNP were determined to be 7.82, 5.69, 3.67, and 0.7 pmol/cm^2^, respectively. From all data collected so far, the catalytic activity of CB[*n*]/AuNPs was found to be dependent on the particle size and CB[*n*] packing density. Thus, we were led to the conclusion that smaller particles with less CB[*5*] exhibit a higher activity due to the reduced blocking of the reactive surface sites [55,56].

## 4. Conclusions

In summary, a series of AuNPs was prepared in situ by CB[*n*]s (*n* = *5*, *6*, *7*, *8*) in basic media. The diameter of the prepared particle was strongly dependent on the ring sizes of CB[*n*]s. The resulting composites could be applied to the catalyzed reduction of 4-NP in the presence of NaBH_4_. The packing densities of CB[*n*]s and free surface area binding site availabilities on CB[*n*]-AuNPs were systematically investigated for the first time. It seems that a bigger macrocyclic ring with more binding units and flexibility could form a stronger bond to the gold surface. However, this needs to be testified by further experiments. We believe that smaller CB[*n*]-capped AuNPs with high aqueous solubilities, high stabilities, uniform sizes, and low densities of capping agents could have important applications in catalysis. Meanwhile, bigger CB[*n*]-capped AuNPs that bear high densities of macrocycles have great potential for use in delivering cargos and constructing supramolecular assembly in a solution or on the surface. This is made possible by virtue of the special inclusion ability of CB[*n*]s.

## Figures and Tables

**Table 1:** TEM determined particle sizes for AuNPs prepared in situ by CB[*n*] ^1^.

AuNPs Composite	Portal Diameter of CB[*n*] (nm) ^2^	Cavity Diameter of CB[*n*] (nm) ^2^	Diameter (nm) ^3^	Diameter (nm) ^4^
CB[*5*]/AuNPs	0.24	0.44	3.6 ± 1.0	4.6 ± 0.7
CB[*6*]/AuNPs	0.39	0.58	10.0 ± 4.4	10.4 ± 1.6
CB[*7*]/AuNPs	0.54	0.73	3.3 ± 1.1	12.4 ± 1.8
CB[*8*]/AuNPs	0.69	0.88	7.7 ± 2.9	15.5 ± 2.5

^1^ Error measurements represent the standard deviation of at least 200 measurements. ^2^ CB[*n*]s were prepared by our lab [15,16,17]. ^3^ Cited from Reference [24]. ^4^ This work.

**Table 2:** Percent of weight lost by TGA, calculated packing density for CB[*n*]/AuNPs, and apparent kinetic constant (*K_app_*) and induction time of 4-NP reduction catalyzed by AuNPs ^1^.

AuNPs Composite	Weight Loss %	Packing Density (molecules/nm^2^)	Coverage (%)	*k_app_* (min^−1^) ^3^	Induction Time (s) ^3^
CB[*5*]/AuNPs	1.08	0.08	1.3	11.55 ± 1.4	ND ^4^
CB[*6*]/AuNPs	4.61	0.97	25.6	7.17 ± 0.34	3.75 ± 0.45
CB[*7*]/AuNPs	4.78	1.04	43.8	5.98 ± 0.17	10.24 ± 1.62
CB[*8*]/AuNPs	5.77	1.38	83.9	1.95 ± 0.09	16.59 ± 0.71
AuNPs-citrate (4 nm) ^2^	N/A ^5^	N/A ^5^	N/A	13.39 ± 1.9	ND ^4^
AuNPs-citrate (16 nm) ^2^	N/A ^5^	N/A ^5^	N/A	7.22 ± 0.35	2.01 ± 0.02

^1^ Error measurements represent the standard deviation of three measurements. ^2^ AuNPs-citrate were prepared by our lab. Please find details in the Supplementary Materials [37]. ^3^ *k_app_* and induction time were determined and calculated according to our reported procedures [37,38]. ^4^ Too fast to be determined. ^5^ Not applicable.

## Supplementary Materials

The following are available online at http://www.mdpi.com/2079-4991/8/12/1015/s1: Figure S1: UV-VIS spectrum and the corresponding photographs of the AuNPs capped with (**a**) CB[*5*]; (**b**) CB[*6*]; (**c**) CB[*7*]; (**d**) CB[*8*]; Figure S2: XRD patterns of CB[*n*]-AuNPs; Figure S3: FTIR spectra of CB[*n*] and AuNPs capped with (**a**) CB[*5*]; (**b**) CB[*6*]; (**c**) CB[*7*]; (**d**) CB[*8*]; Figure S4: (**A**) TGA curves of (**a**) CB[*5*]; (b) CB[*6*]; (c) CB[*7*]; (d) CB[*8*]; (**B**) TGA curves of the AuNPs capped with (**a**) CB[*5*]; (**b**) CB[*6*]; (**c**) CB[*7*]; (**d**) CB[*8*]; Figure S5: First-order rate constant plot of 4-NP reduction for (**a**) CB[*5*]/AuNPs; (**b**) CB[*6*]/AuNPs; (**c**) CB[*7*]/AuNPs; (**d**) CB[*8*]/AuNPs; (**e**) Citrate-capped AuNPs (4 nm); (**f**) Citrate-capped AuNPs (16 nm); Figure S6: UV-VIS spectra and first-order rate constant plot of 4-NP reduction for (**a**) and (**d**) CB[*5*]; (**b**) and (**e**) CB[*6*]; (**c**) and (**f**) CB[*7*]; Figure S7: (**c**) Dependence of the apparent rate constant, *k_app_*, on the concentration of 4-NP at constant concentrations of BH_4_^−^ (21 mM in (**a**), 10.5 mM in (**b**)); (**f**) Dependence of the apparent rate constant, *k_app_*, on the concentration of BH_4_^−^ at constant concentrations of 4-NP (0.075 mM in (**d**), 0.05 mM in (**e**)); Figure S8: Partial ^1^H NMR spectra (600 MHz, D_2_O) of (**a**) benzimidazole; (**b**) benzimidazole and CB[*6*]; (**c**) benzimidazole and CB[*7*]; and (**d**) benzimidazole and CB[*8*]; Figure S9: 2-MBI and adenine adsorption experiments; (**a**) UV-VIS spectra of 2-MBI in the mixture of 2-MBI and amantadine (1:1 molar ratio, 50 μM) after 24 h alone (**A**), or treatment with CB[*5*]/AuNPs (**B**), CB[*6*]/AuNPs (**C**), CB[*7*]/AuNPs (**D**) and CB[*8*]/AuNPs (**E**); (**b**) UV-VIS spectra of adenine in the mixture of adenine and amantadine (1:1 molar ratio, 50 μM) after 24 h alone (**A**), or treatment with CB[*5*]/AuNPs (**B**), CB[*6*]/AuNPs (**C**), CB[*7*]/AuNPs (**D**) and CB[*8*]/AuNPs (**E**).
