# Supplementary material for: Controllable Synthesis and Catalytic Performance of Gold Nanoparticles with Cucurbit[n]urils (n = 5–8)
Source: Nanomaterials (Basel). 2018 Dec 6;8(12):1015. doi: 10.3390/nano8121015 (PMC6316165; doi:10.3390/nano8121015)
Supplement: Supplementary file 1 [file nanomaterials-08-01015-s001.pdf]

Supporting Information for

Controllable Synthesis and Catalytic Performance of Gold Nanoparticles with Cucurbit[n]urils (n = 5-8)

Liangfeng Zhang, Simin Liu, Yuhua Wang, Haijun Zhang and Feng Liang*

The State Key Laboratory of Refractories and Metallurgy, Coal Conversion and New Carbon Materials Hubei Key Laboratory, Hubei province Key Laboratory of Science in Metallurgical Process, School of Chemistry and Chemical Engineering, Wuhan University of Science and Technology, Wuhan430081,China.

Packing density of CB[n]on the gold particle surface

CB[n]/AuNPs were modelled as spheres with an average radius (r) provided by TEM. Utilizing the weightfraction of CB[n] ($w_{CB[n]}$), the weight fractionof the AuNP core (w_{Au}), the mass of an individualAuNP (m_{Au}), the relative molecular mass of CB[n] ($M_{CB[n]}$), the surface area of an individual Au nanoparticle (S_{Au}), the occupied area of one CB[n] molecule($S_{CB[n]}$), $N_A$is Avogadro constant, the packing density of CB[n] molecules (SC) and coverage could be calculated using equations below.

$$\mathrm{SC} = \frac{m_{Au} \times w_{CB[n]} \times N_A}{w_{Au} \times M_{CB[n]} \times S_{Au}}$$

$$\mathrm{Coverage} = \mathrm{SC} \times S_{CB[n]} \times 100\%$$

The first weight loss of CB[n] samples up to 300 ºC in the TGA could be attributed to the release of physically absorbed water and chemical decomposition of the cucurbiturils was observed above 370 ºC [1,2]. From TG curves, the weight fraction ofCB[5], CB[6], CB[7]andCB[8] were 1.08, 4.61, 4.78, and 5.77%, respectively. In the representative case of CB[7], the average size of the CB[7]/AuNPs is 12.4 nm, the mass and surface area of an individual CB[7]/AuNP particle are 1.93×10^{-17} g and 482.8 nm^2. Therefore, the total mass of CB[7] on the surface of an individualCB[7]/AuNP is 9.69×10^{-19} g. The molecular weight of CB[7] is 502. Thus, the packing density of CB[7] is 1.04 molecules/nm^2 and the occupied area of one CB[7] molecule is 0.418 nm^2based on the diameter of 0.73 nm, and the coverage of CB[7] on CB[7]/AuNP is 43.5%.

References:

[1] Bardelang D,Udachin KA, Leek DM,Margeson JC, Chan C, Ratcliffe CI, Ripmeester JA (2011) Cucurbit[n]urils (n = 5–8): A Comprehensive Solid State Study. Cryst Growth Des 11:5598–5614

[2] Yoon M, Suh K, Kim H, Kim Y,Selvapalam N (2011) High and Highly Anisotropic Proton Conductivity in Organic Molecular Porous Materials. Angew Chem Int Ed 50:7870–7873

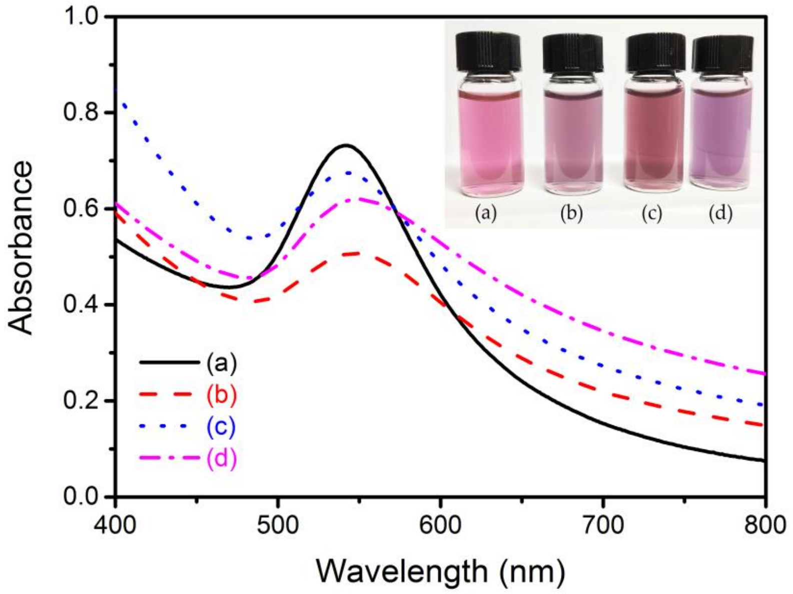

Fig S1. UV-vis spectrum and the corresponding photographs of theAuNPs capped with (**a**) CB[5]; (**b**) CB[6]; (**c**) CB[7]; (**d**) CB[8].

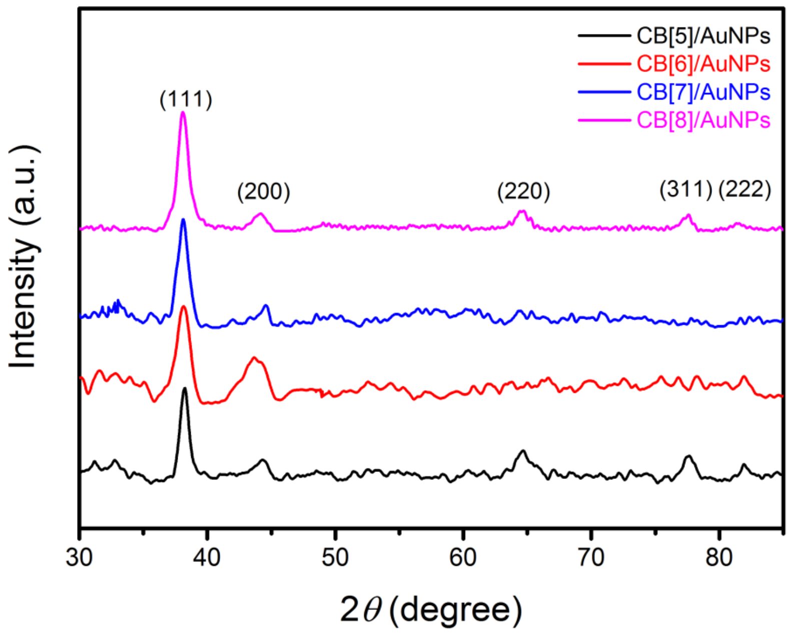

Fig S2. XRD patterns of CB[n]-AuNPs.

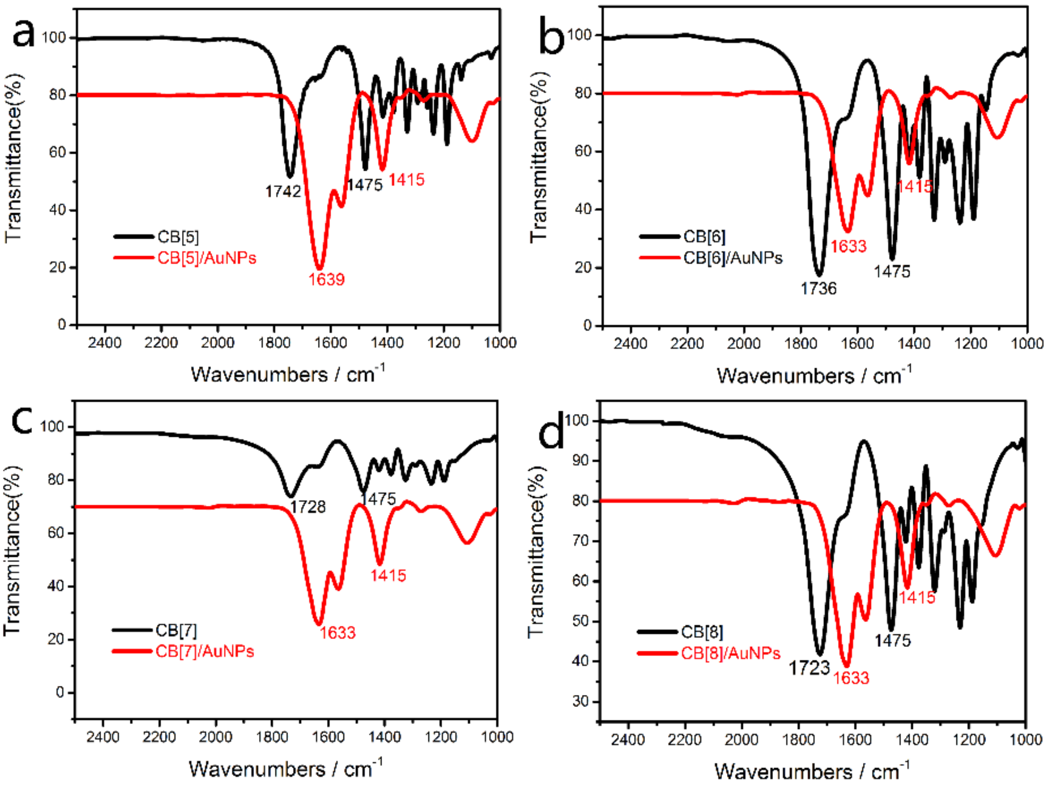

Fig S3. FT-IR spectra of CB[n] and AuNPs capped with (**a**) CB[5]; (**b**) CB[6]; (**c**) CB[7]; (**d**) CB[8].

A

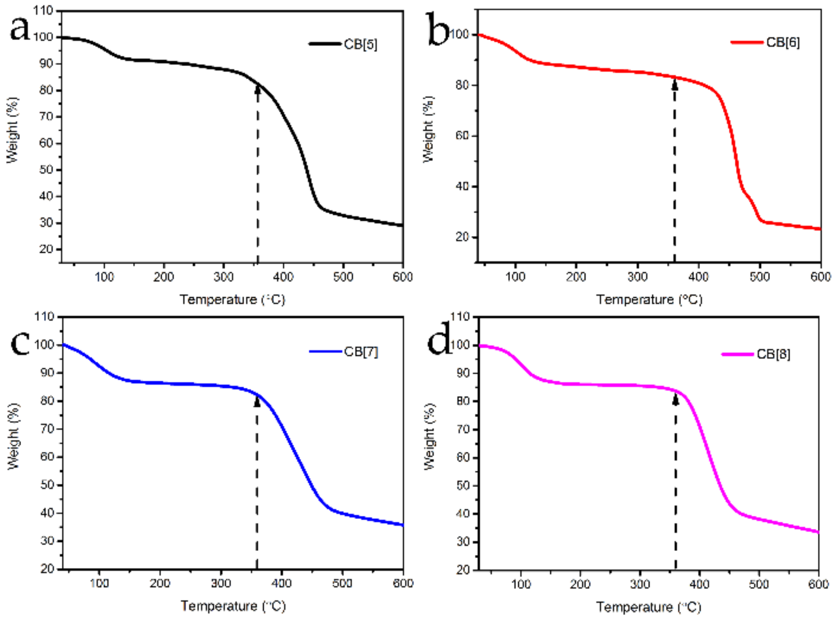

B

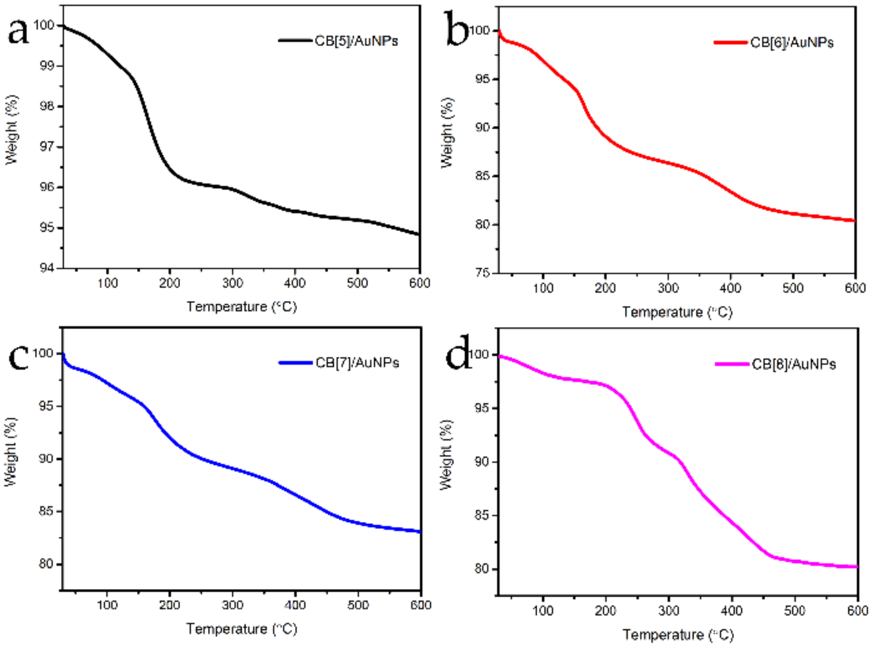

Fig S4. (**A**) TGA curves of (**a**) CB[5]; (**b**) CB[6]; (**c**) CB[7]; (**d**) CB[8]; (**B**)TGA curves of theAuNPs capped with (**a**) CB[5]; (**b**) CB[6]; (**c**) CB[7]; (**d**) CB[8].

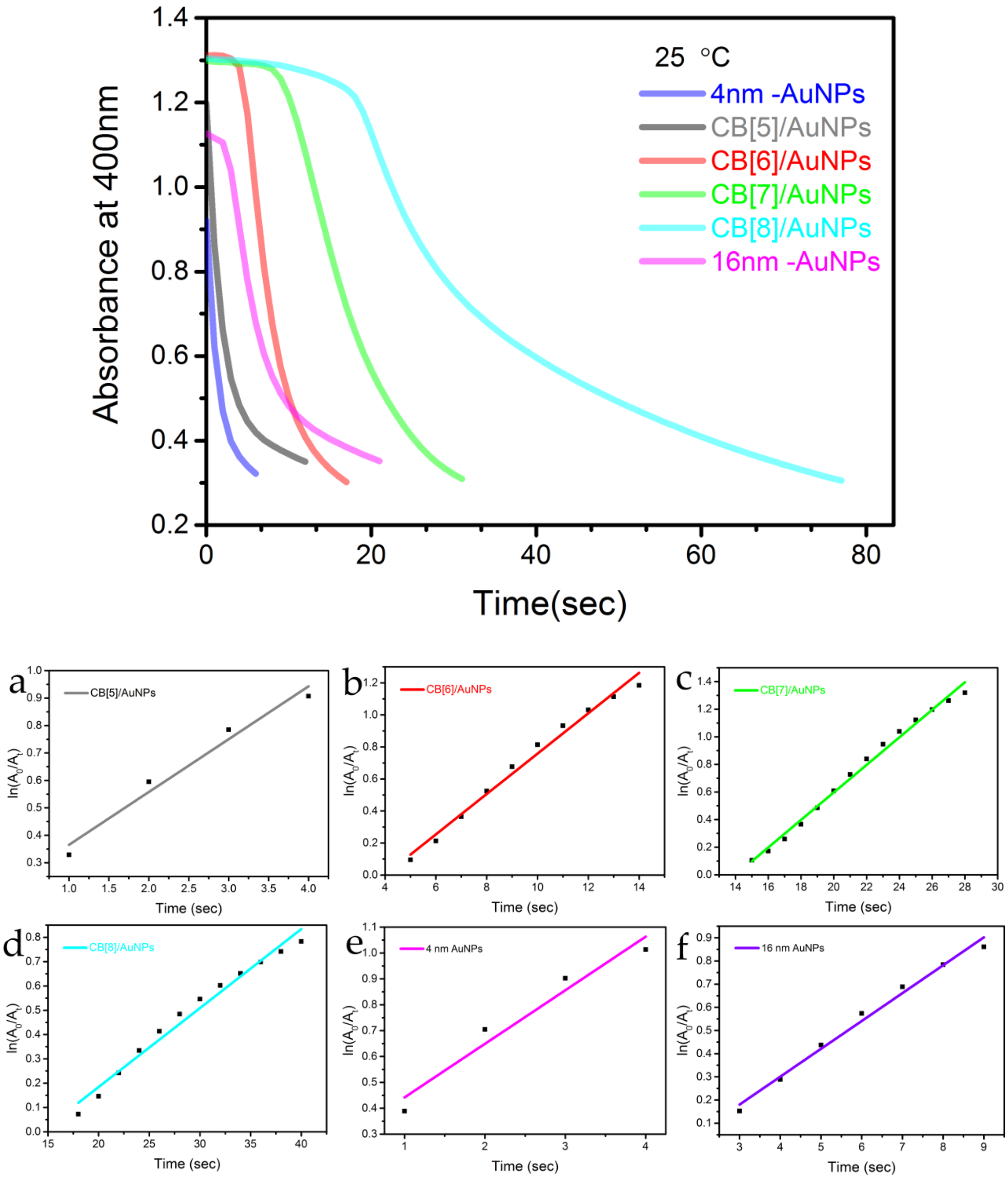

Fig S5. First order rate constant plot of 4-NP reductionfor (**a**) CB[5]/AuNPs; (**b**) CB[6]/AuNPs; (**c**) CB[7]/AuNPs; (**d**) CB[8]/AuNPs; (**e**) Citrate-capped AuNPs (4 nm); (**f**) Citrate-capped AuNPs (16 nm).

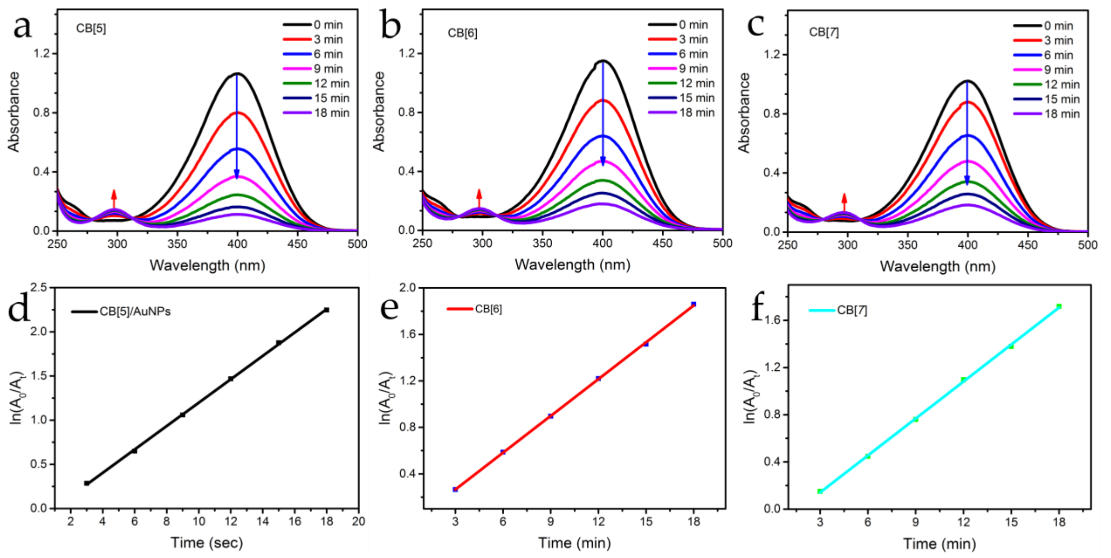

Fig S6. UV-vis spectra and first order rate constant plot of 4-NP reduction for (**a**) and (**d**) CB[5]; (**b**) and (**e**) CB[6];(**c**) and (**f**) CB[7].

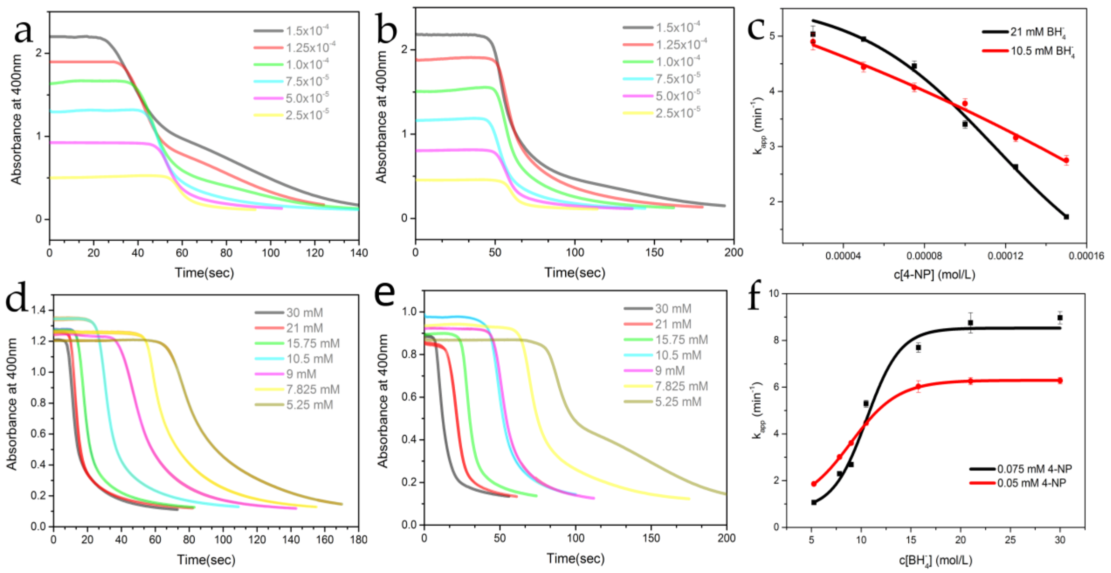

Fig S7. (**c**) Dependence of the apparent rate constant, k_{app}, on the concentration of 4-NP at constant concentrations of BH_4^- (21 mM in (**a**), 10.5 mM in (**b**)); (**f**) Dependence of the apparent rate constant, k_{app}, on the concentration ofBH_4^- at constant concentrations of 4-NP (0.075 mM in (**d**), 0.05 mM in (**e**)).

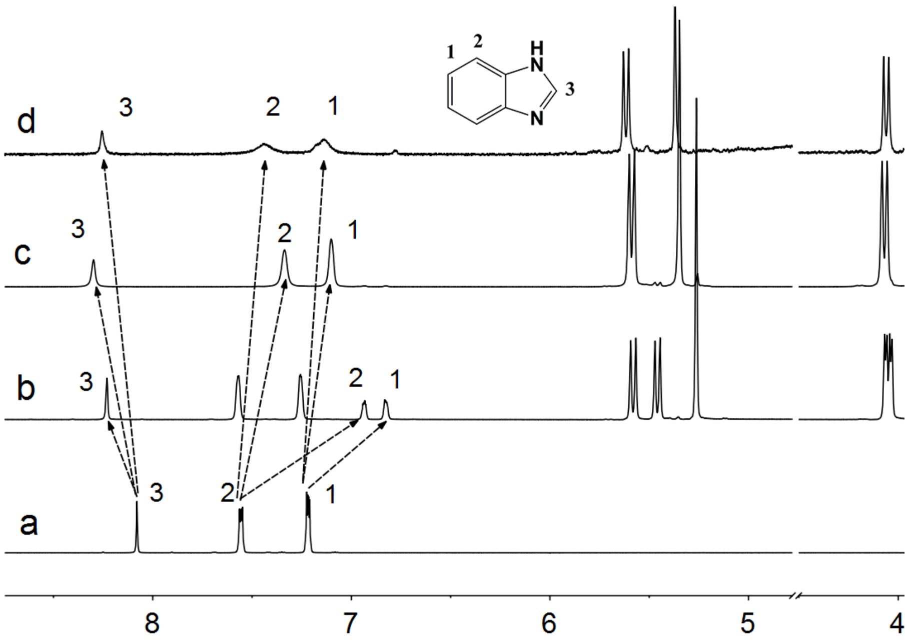

Fig S8. Partial ^{1}H NMR spectra (600 MHz, D_2O) of (**a**) benzimidazole; (**b**) benzimidazole and CB[6]; (**c**)benzimidazole and CB[7]; and (**d**)benzimidazole and CB[8].

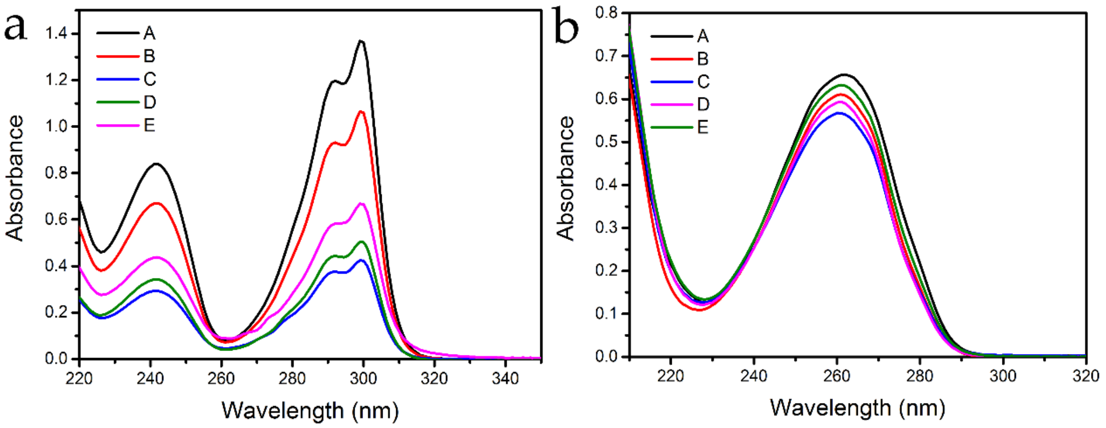

Fig S9. 2-MBI and adenine adsorption experiments. (**a**) UV-vis spectra of 2-MBI in the mixture of 2-MBI and amantadine (1:1 molar ratio, 50μM) after 24 h alone (**A**), or treatment with CB[5]/AuNPs (**B**), CB[6]/AuNPs(**C**), CB[7]/AuNPs(**D**) and CB[8]/AuNPs (**E**). (**b**) UV-vis spectra of adenine in the mixture of adenine and amantadine (1:1 molar ratio, 50μM) after 24 h alone (**A**), or treatment with CB[5]/AuNPs(**B**), CB[6]/AuNPs (**C**), CB[7]/AuNPs (**D**) and CB[8]/AuNPs (**E**).
